# Essential Palatal Tremor Managed by Cognitive Behavioral Therapy

**DOI:** 10.1155/2015/414620

**Published:** 2015-12-10

**Authors:** Tomohisa Kitamura, Tsuyoshi Sato, Naoki Hayashi, Yosuke Fukushima, Tetsuya Yoda

**Affiliations:** Department of Oral and Maxillofacial Surgery, Saitama Medical University, 38 Moro-hongou, Moroyama-machi, Iruma-gun, Saitama 350-0495, Japan

## Abstract

*Background*. Essential palatal tremor is a disorder of unknown etiology involving involuntary movement of the uvula and soft palate. Treatment attempts including drugs or surgery have been conducted to cease the rhythmical movement.* Case Report*. A 55-year-old female visited our department complaining of a sudden, noticeable, intermittent, and rhythmical clicking noise in her throat for five years. Oral examination revealed rhythmical contractions of the soft palate with clicking at the frequency of 120 per min. Magnetic resonance imaging (MRI) examination of the brain performed after consulting with the department of neuropathic internal medicine showed no abnormalities. Thus, essential palatal tremor was diagnosed. The symptoms improved with cognitive behavioral therapy without drugs or surgical treatments. The patient is now able to stop the rhythmical movement voluntarily.* Discussion*. Cognitive behavioral therapy might be suitable as first-line therapy for essential palatal tremor because the therapy is noninvasive.

## 1. Introduction

Palatal tremor is characterized by rhythmical movement of the soft palate with clicking noises and muscle spasms [1]. It is classified into two subtypes [2]. Pearce reported that symptomatic palatal tremor, which is caused by a lesion in the triangle of Guillain and Mollaret, presents with the movement of the levator veli palatini muscles accompanied by ear clicking and frequently occurs during sleep [3]. In contrast, essential palatal tremor exhibits the movement of the tensor veli palatini in the absence of any brainstem lesions or physical or radiological signs. Treatment with drugs or surgery has been attempted to cease the rhythmical movement in essential palatal tremor [2]. We here report a case of essential palatal tremor where the symptoms were improved by cognitive behavioral therapy without drugs or surgical treatments.

## 2. Case Presentation

A 55-year-old female visited our department complaining of intermittent and rhythmical clicking in the throat for five years. Although she noticed a clicking noise, she could not recognize the origin of the sound. She found the clicking uncomfortable because the sudden sound from her throat was noticeable in public. Oral examination revealed rhythmical contractions of the soft palate with clicking at the frequency of 120 per min (Video 1 in Supplementary Material available online at http://dx.doi.org/10.1155/2015/414620). Her past medical history included cervical spinal canal stenosis and a menopause. She was not taking any medications regularly. Magnetic resonance imaging (MRI) examination of the brain performed after consulting with the department of neuropathic internal medicine revealed no abnormalities. Furthermore, the rhythmical movement which appeared irregularly did not occur during sleep. Thus, essential palatal tremor was diagnosed.

Although a prescription of clonazepam was considered, the patient denied receiving any medications and refused to undergo other pharmacological and surgical treatments. Therefore, we provided cognitive behavioral therapy to stop the rhythmical clicking. We guided her as a cognitive behavioral therapy to breathe deeply and produce her “Ah” voice when her involuntary palatal tremor occurred. Expectedly, the rhythmical clicking stopped for approximately five seconds after deep breathing and the phonation of “Ah” (Video 2).

Her symptoms significantly improved after 16 months, and she was able to stop the rhythmical movement voluntarily.

## 3. Discussion

In this report, we described a patient suffering from essential palatal tremor whose symptoms were improved by cognitive behavioral therapy without surgical treatments or medications. The occasional and sudden rhythmical movement can now be stopped voluntarily by the patient.

Several therapies including surgical and pharmacological treatments have been attempted to stop the clicking in essential palatal tremor [3–5]. Although many surgical procedures have been described, there are no commonly successful surgical treatments [2]. Various drugs such as cholinergic drugs, benzodiazepam, anti-Parkinsonism drugs, anticonvulsants, relaxants, 5-hydroxytryptophan, levodopa, and lithium are usually prescribed as treatment [6]. Treatment with botulinum toxin has also been reported to be effective [7, 8], but attention should be paid to the adverse side effects such as neuromuscular inhibition [9]. In this case, we considered the usage of botulinum toxin; however, in Japan the prescription of the drug is limited to “off-label” use under health insurance.

Jacobs et al. reported a patient who could voluntarily stop the clicking. Yokota et al. reported disappearance of clicking after explaining to the patient that the disease was benign and transient, suggesting that cognitive behavioral therapy, with a goal-oriented practical approach to problem solving, is useful for the treatment of essential palatal tremor [10, 11]. Psychomotor influence on essential palatal tremor has been observed in another case where the clicking was stopped when the patient was calculating [11]. In our case, the patient was able to voluntarily stop the clicking after deep breathing and the phonation of “Ah.” This case suggested that cognitive behavioral therapy should be considered one of the therapies for essential palatal tremor.

## Supplementary Material

Video 1, Rhythmical contractions of the soft palate with clicking.
Video 2, A cognitive behavioral therapy to breathe deeply and produce her “Ah” voice.
